# Silent Myocardial Ischemia:A challenge for the doctors

**DOI:** 10.12669/pjms.301.4685

**Published:** 2014

**Authors:** Zhoupeng Wu, Jichun Zhao

**Affiliations:** 1 Zhoupeng Wu, Department of Vascular Surgery, West China Hospital, Sichuan University, China.; 2 Jichun Zhao, Department of Vascular Surgery, West China Hospital, Sichuan University, China.

Silent myocardial ischemia (SMI) is an asymptomatic coronary syndrome without a history of infarction, and is associated with a higher risk of cardiovascular events.^1^ SMI is usually detected incidentally during routine controls. Although there is a high prevalence in elderly female patients, routine screening is not recommended^1^.As in this current case, in patients with SMI, in addition to aspirin, statin, and angiotensin receptor blockers, beta blocker therapy should be considered in the treatment.^2^ The routine use of proton pump inhibitors is not recommended in patients taking daily doses of aspirin.^2^

The contemporary incidence of left ventricular (LV) thrombus after acute MI is approximately 5 %, and systemic embolization in patients with LV thrombus is mostly associated with decreased LV ejection fraction and LV aneurysm.^3^ Most episodes of systemic embolization occur in the first two weeks after acute MI^3^. In patients with multiple systemic embolic infarctions, further investigation including cardiac evaluation with transthoracic echocardiography (TTE) as an initial diagnostic test should be carried out to detect an embolic origin.^4^ Transesophageal echocardiography (TEE) is suggested to perform in clinical conditions when there is a question about the information obtained using TTE. TEE is especially recommended in identification of cardioembolic source, assessment of left atrial appendage, and intra cardiac masses.^5^ Anticoagulation with warfarin sodium should be prescribed to patients found to have an LV thrombus or embolic phenomenon. Prophylactic anticoagulation could be considered even in patients with LV dysfunction and extensive wall motion abnormalities that do not have a thrombus.^4^ Actually, about the necessity of TEE and anticoagulant therapy in patients with cryptogenic MI, in this current case, there was no clear evidence about the association between multiple systemic embolization and MI.

In conclusion, in patients with systemic embolization, there is no doubt that TEE should be performed even in clinical suspicion of cardio embolic source, and anticoagulant therapy should be considered as an acceptable option for initial therapy whatever the cause is.
